# Luminescent Transparent Wood for Diffusive White Light Generation

**DOI:** 10.1021/acsaom.6c00236

**Published:** 2026-06-17

**Authors:** Francesco Bolognesi, Seyed Ehsan Hadi, Vivek Richards Pakkam Gabriel, Ravi Shanker, Yuanyuan Li, Lars A. Berglund, Hui Chen, Ilya Sychugov

**Affiliations:** † Department of Fibre and Polymer Technology, 7655 KTH Royal Institute of Technology, Teknikringen 56, Stockholm 10044, Sweden; ‡ Department of Enterprise Engineering “Mario Lucertini”, 9318 University of Rome “Tor Vergata”, Via del Politecnico, 00133 Rome, Italy; § Department of Applied Physics School of Engineering Sciences, KTH Royal Institute of Technology, Hannes Alfvens väg 12, Stockholm 11419, Sweden

**Keywords:** transparent wood, diffusive white light, quantum dots, photoluminescence, quantum yield

## Abstract

Diffused white light is generated from a single-layer transparent wood (TW) composite incorporating a mixture of green and red CdSe/ZnS quantum dots (QDs) under blue light-emitting diode (LED) excitation. Unlike conventional multilayer or single-emitter systems, a simple codispersion strategy is used in which multiple QDs are incorporated within a single TW layer. Notably, no blue-emitting QDs are required. The QD-TW composites show excellent mechanical properties. The 0.6 mm thick composite shows high optical transmittance, strong haze, and a quantum yield of 42%. Under UV-blue excitation, the composite produces homogeneous white light through the combined contributions of QD emission and TW light scattering. TW can serve as a sustainable, mechanically robust platform for multicolor emitters and provide glare-free, uniform illumination.

## Graphical abstract

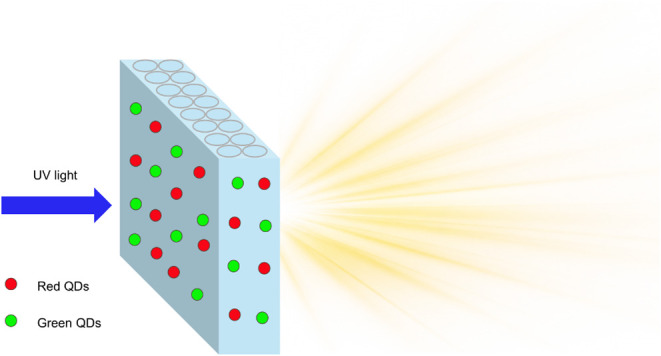

## 1 Introduction

Environmentally friendly and energy-efficient white light-emitting diodes (W-LEDs) have become a key technology for next-generation lighting. A typical W-LED device consists of an LED chip, a chromatic conversion material, and a polymeric encapsulation layer.^1,2^ A widely adopted approach is based on blue LED chips combined with color conversion materials to generate white light.^1,3^ In this context, quantum dots (QDs) have attracted increasing attention as color conversion materials owing to their size-tunable emission, narrow spectral line widths, and high color purity.^4–8^ Advanced QDs synthesis strategies, including alloy engineering, shell passivation, and ion-exchange-assisted growth, have further improved their optical performance by suppressing defect states, narrowing emission line widths, and enhancing photoluminescence quantum yields, thereby improving their suitability for light-emitting applications.^9–11^

Despite these advantages, QDs are inherently sensitive to oxygen, moisture, and thermal stress, which can lead to photoluminescence degradation. To ensure operational stability, QDs are typically embedded within protective matrices. While effective, conventional polymer matrices, such as epoxy resins, provide basic encapsulation but are derived from fossil resources and have the mechanical property limitations.^12,13^ Therefore, alternative encapsulation materials that are combine biobased origin with improved mechanical performance are of considerable interest.

Transparent wood (TW), obtained by removing lignin or bleaching of wood substrates followed by infiltration with a refractive-index-matched polymer, resulting in a hierarchical structure that preserves the wood microarchitecture while enabling efficient light transport.^14^ TW has recently emerged as a class of sustainable optical materials. This combination of high optical transmittance (over 90% at 1.5 mm thickness),^15^ mechanical robustness (up to 270 MPa),^16^ and low thermal conductivity^17^ makes TW a promising biobased platform for sustainable photonic and structural applications, including lighting materials,^18,19^ smart windows,^20^ and photoelectric devices.^21–24^

Recent advances in TW-based luminescent composites have explored several strategies for integration of QDs. Multilayer TW architectures, in which different emitters are spatially separated, have been developed to achieve color mixing and white light emission under external excitation. For example, laminated structures incorporating red and green QDs in distinct TW layers have demonstrated effective color conversion;^21,24^ however, such approaches typically require complex fabrication processes and precise layer engineering. In many cases, these systems also rely on additional blue-emitting components (blue QDs), which may be unnecessary for white light generation if the light source is a blue LED itself. In contrast, single-layer TW systems incorporating QDs offer a simpler structure and have been demonstrated in several studies. For instance, QDs dispersed in polymer-infiltrated wood matrices have enabled uniform luminescence with tunable emission colors.^18,25,26^ However, these systems are often limited to single-emitter configurations, resulting in monochromatic output. To achieve white light emission, mixtures of multiple emitters have also been explored. Previous studies have demonstrated single-layer TW composites incorporating multiple carbon QDs,^2,12^ producing white light under UV excitation. Nevertheless, these approaches again rely on the inclusion of blue-emitting components (blue QDs), which may be unnecessary when suitable blue light is applied for excitation. In some cases, poly­(acrylic acid) has been used as the matrix, whose high hygroscopicity makes it unsuitable for engineering applications.^12^ In another study, Chung et al. prepared yellow QD-doped PMMA layers via polymerization of MMA/QD mixtures on blue LED chips to achieve white light emission;^27^ however, the mechanical properties are much lower than for QD-TW. These studies highlight the potential of QD-TW composites for white light-emitting applications, while also indicating that achieving efficient white light emission in a single-layer TW system with a simple structure and minimal emitter complexity remains a significant challenge.

In the present work, we demonstrate that efficient white light emission can be achieved in a single-layer TW system by incorporating green and red QDs into a wood-polymer composite. Unlike most previous approaches that rely on spatially separated QDs in multilayer structures, this work focuses on codispersing green and red QDs within a single TW layer, enabling volumetric color mixing and uniform light redistribution within a single scattering medium. Importantly, this approach eliminates the need for blue-emitting QDs. Instead, the blue excitation light is directly utilized as one component of the final white light, while red and green emissions are provided by the embedded QDs. This reduces material complexity and avoids unnecessary spectral overlap or reabsorption associated with additional emitters. Emphasis is placed on achieving uniform QD dispersion to preserve photoluminescence efficiency and maintain the mechanical integrity of the material. Benefiting from the high transmittance and intrinsic light-scattering properties of TW, efficient excitation of QDs together with homogeneous photon redistribution can be achieved throughout the composite thickness, enabling low-glare white light generation. Rather than targeting conventional display technologies, this work highlights TW as a sustainable photonic material for applications such as decorative lighting, smart optical panels, and large-area diffuse emitters.

## 2 Materials and Methods

### 2.1 Materials

Radial Aspen veneers (*Populus tremula* L. density: 410 kg/m^3^, purchased from Holm Trävaror AB) with dimensions of 20 × 20 × 0.6 mm^3^ were used as the wood substrate. Glacial acetic acid (CH_3_COOH), sodium acetate (C_2_H_3_NaO_2_), sodium chlorite (NaClO_2_), methyl methacrylate (MMA), and the initiator 2,2′-Azobis (2-methyl propionitrile) (AIBN) were purchased from Sigma-Aldrich. CdSe quantum dots (QDs) coated with a ZnS shell were used for improved stability. The QDs were surface functionalized with a mixture of oleic acid, oleylamine, and dodecanethiol to enhance dispersion. Emission peaks were centered at approximately 525 nm (green, smaller CdSe core) and 625 nm (red, larger CdSe core). The QDs were purchased from Mesolight Inc. at a concentration of 25 mg/mL.

### 2.2 Wood Delignification

Wood veneers were chemically delignified using 1 wt % of NaClO_2_ in an acetate buffer solution (pH 4.6).^19^ The treatment was carried out at 80 °C for 2 h until the samples turned white. The delignified samples were thoroughly washed with deionized water to remove residual chemicals, followed by solvent exchange using ethanol and acetone for dehydration. The mass loss after delignification was determined using oven-dried samples (105 °C) by comparing the mass before and after treatment.

### 2.3 Prepolymerization of Methyl Methacrylate

The prepolymerization of MMA was conducted at 75 °C for approximately 15 min in a round-bottom flask using 0.3 wt % of AIBN as the initiator. The reaction was subsequently quenched by cooling the solution to room temperature using an ice–water bath, yielding prepolymerized MMA (pre-MMA).

### 2.4 Transparent Wood Fabrication

Green (525 nm), red (625 nm) and a mixed green/red (1:1 by weight) CdSe/ZnS QD solutions were diluted in pre-MMA to a final concentration of 0.125 mg/mL. Delignified wood samples were then immersed in the QD/pre-MMA solutions and subjected to vacuum-assisted impregnation. The impregnated samples were subsequently sandwiched between glass slides, wrapped in aluminum foil, and thermally cured at 70 °C for 4 h to complete polymerization. This resulted in transparent wood (TW), green-emitting TW (G-TW), red-emitting TW (R-TW), and mixed QD TW (M-TW), all with a thickness of approximately 0.6 mm. The QD content in the composite *N*_QD_ was calculated according to

1 $$N_{\mathrm{QD}}=\frac{V_{m}\times {\mathrm{QDs}}_{\mathrm{conc}}}{m_{\mathrm{comp}}}$$

where *V*_m_ is the volume of PMMA in the composite, QDs_conc_ is the initial concentration of QDs in the pre-MMA, and *m*_comp_ is the mass of the composite.

### 2.5 SEM Characterization

Cross sections of native and delignified wood were prepared by freeze-fracture in liquid nitrogen (−196 °C), followed by freeze-drying at −110 °C for at least 12 h. Prior to imaging, samples were sputter-coated with Pt/Pd (Cressington R208, U.K.). Cross sections of TW and R-TW were prepared using an ultramicrotome equipped with a diamond knife (Leica ARTOS 3D, DiATOME (trim 20) knife). Morphology was examined using a field-emission scanning electron microscope (FE-SEM, Hitachi S-4800, Japan) at an accelerating voltage of 1 kV in secondary electron mode. In addition, uncoated cross sections of R-TW were analyzed using a tabletop SEM (Hitachi TM3000) operated at 15 kV in backscattered electron mode to enhance contrast from QD-rich regions.

### 2.6 Wood Volume Fraction

The wood volume fraction (*V*_f_) in the composite was calculated as

2 $$V_{f}=\frac{W_{f}\times \rho_{c}}{\rho_{f}}$$

where *W*_f_ is the mass fraction of the wood template, ρ_c_ is the composite density, and ρ_f_ is the density of cellulose (1550 kg/m^3^).

### 2.7 Mechanical Testing

Tensile tests were performed on PMMA and TW specimens (50 × 5 × 0.6 mm^3^) using an Instron E1000 equipped with a 2 kN load cell and a video extensometer. Tests were conducted at a strain rate of 10%/min with a gauge length of 20 mm.

### 2.8 Optical Properties

Optical transmittance and haze were measured for 0.6 mm thick TW specimens using an integrating sphere setup in accordance with ASTM D1003,^28^ with modifications for highly scattering materials as described in previous work.^29^ A laser-driven xenon plasma light source (EQ-99 from Energetiq Technology Inc.) coupled with a tunable monochromator (Princeton Instruments) was used. The incident beam (diameter 6 mm) was directed through the sample into the integrating sphere (diameter 150 mm) via the input port (diameter 13 mm). Transmitted light was collected via an optical fiber and analyzed using a spectrometer equipped with a cooled CCD detector. For the haze measurement, the exit port with a diameter of 13 mm was selected according to the beam size and integrating sphere size. And haze was calculated according to

3 $$\mathrm{haze}=\left(\frac{T_{4}}{T_{2}}-\frac{T_{3}}{T_{1}}\right)\times 100$$

where *T*_1_-*T*_4_ correspond to different measurement configurations as defined in the standard.^28^

Photoluminescence (PL) spectra and quantum yield (QY) were measured using the same integrating sphere setup. Excitation wavelengths of 395, 410, 430, and 450 nm were used. The QY was calculated as

4 $$\mathrm{QY}=\frac{{\int F}_{\mathrm{emitted}}d\lambda }{{\int F}_{\mathrm{absorbed}}d\lambda }$$

where ∫*F*_emitted_ is the integrated emitted photon flux and ∫*F*_absorbed_ is the integrated absorbed excitation photon flux. Reported values represent averages of three samples, with an estimated relative systematic error of ∼10%.

Time-resolved photoluminescence (TRPL) measurements were performed to investigate the recombination dynamics of the QD-TW composites. The samples were excited using a pulsed diode laser (λ = 405 nm), and the emission decay signals were collected using a time-correlated single-photon counting system. The decay curves were fitted using a stretched exponential decay model

5 $$y=y_{0}+A\times \exp\left(-{\left(\frac{t}{\tau }\right)}^{\beta }\right)$$

where τ is the characteristic lifetime and β is the stretching factor describing the distribution of recombination environments within the composite matrix. Color-specific decay curves in the mixed composite (M-TW) were obtained using green/red bandpass filters.

The photostability of the QD-TW composites was evaluated under continuous UV irradiation using a UV lamp (UVP, Inc. B-100Y) with a power density of approximately 30 mW/cm^2^. The samples were continuously exposed to UV light, and the photoluminescence QY was measured every 30 min using an excitation wavelength of 395 nm. The QY evolution was monitored to evaluate the stability of the composites under prolonged UV exposure conditions.

The CIE color coordinates were obtained by the multiplication of the spectral power at each wavelength (measured transmittance spectra under UV excitation light) times the weighting factor from each of the eye three-color matching functions, then summing these contributions gives tristimulus values. The correlated color temperature (CCT) was subsequently calculated from the obtained CIE chromaticity coordinates.

## 3 Results and Discussion

### 3.1 Processing and Microstructure

Figure 1 illustrates the transformation of natural wood into transparent and luminescent composites through delignification followed by impregnation with QD/pre-MMA mixtures. The removal of lignin reduces light absorption, while polymer infiltration decreases refractive index mismatch and associated light scattering in the wood structure.^30,31^

**Figure 1:**
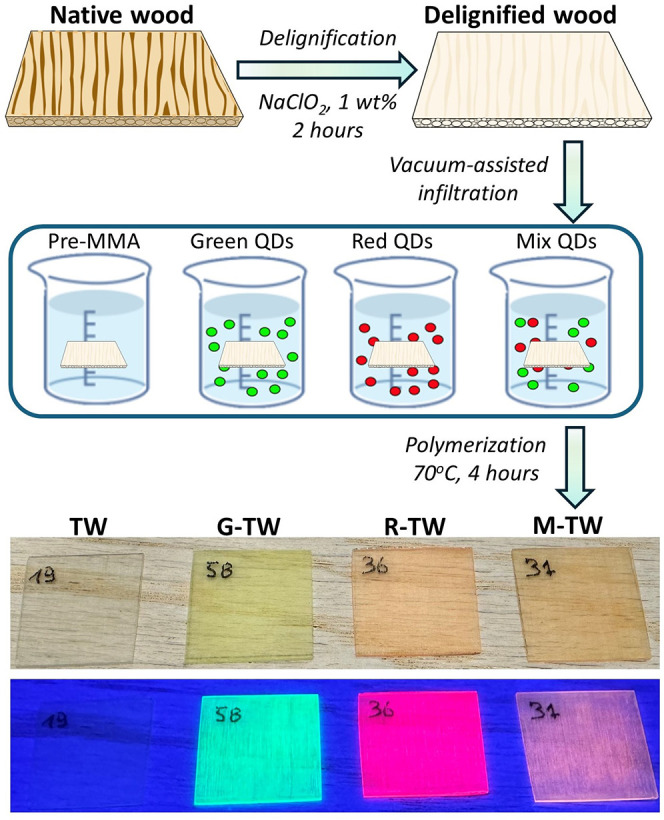
Schematic representation of neat and luminescent transparent wood fabrication procedure and their appearance in natural daylight and UV light. Red and green quantum dots were mixed into the resin solution. TW: a transparent wood composite without quantum dots, G-TW: contains green QDs, R-TW: contains red QDs and M-TW: contains a mixture of red and green QDs.

After impregnation with pre-MMA containing different QDs, three types of luminescent TW composites were obtained: G-TW (green QDs), R-TW (red QDs), and M-TW (mixed green and red QDs), with neat TW serving as a reference. Under ambient light, all samples appear transparent, whereas under UV illumination they exhibit distinct luminescent behavior. G-TW and R-TW show characteristic green and red emission, respectively, while M-TW exhibits combined emission, resulting in an orange appearance.

Microstructural images and corresponding sample photographs are presented in Figure 2. Native aspen wood (Figure 2a_1_) exhibits a pale brown color due to chromophoric components such as lignin. Following delignification, these components are removed, resulting in a white appearance (Figure 2b_1_), where light scattering dominates.

**Figure 2:**
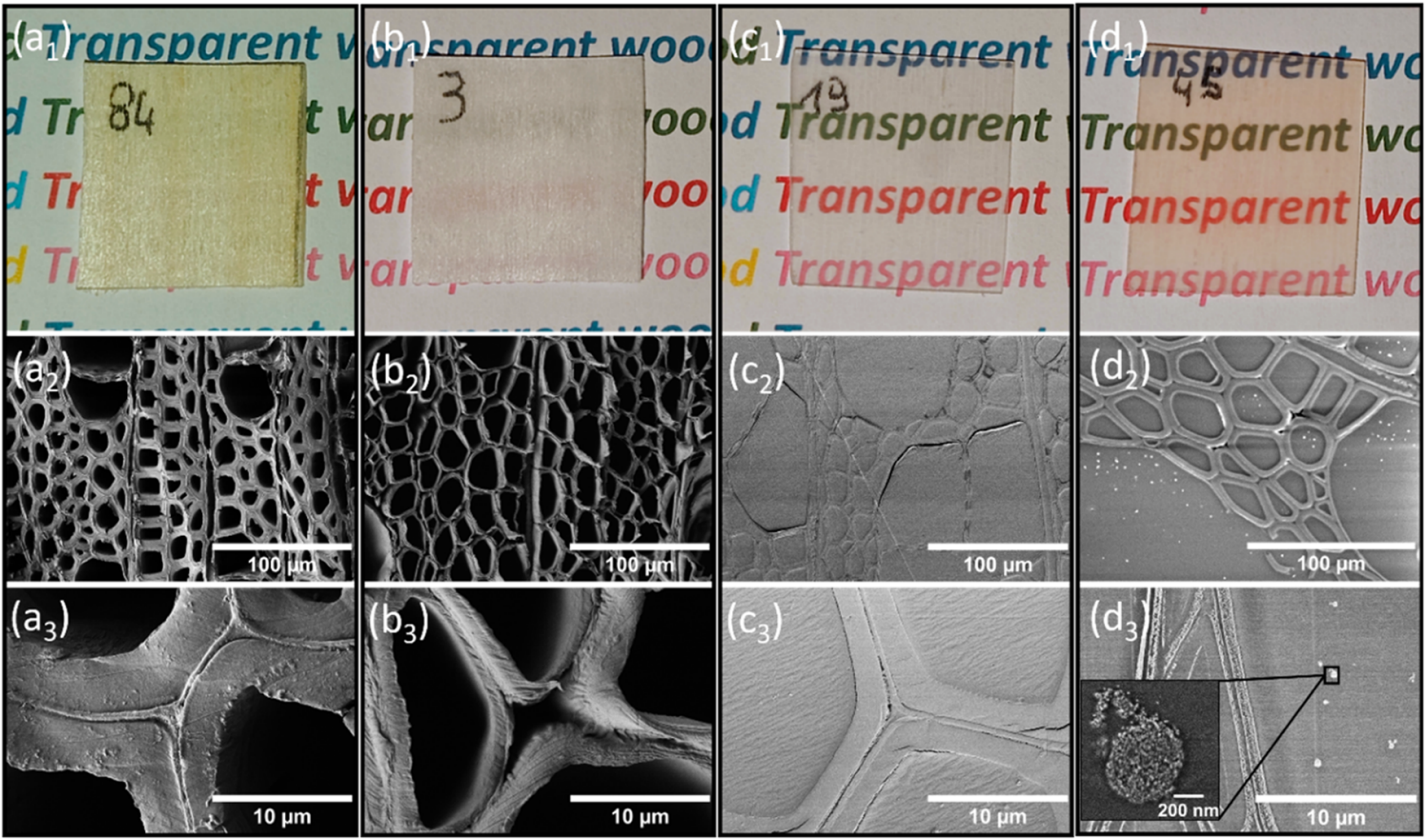
Macro images of (a_1_) native aspen wood, (b_1_) delignified aspen wood, (c_1_) TW, and (d_1_) R-TW. SEM micrographs of (a_2_, a_3_) native aspen wood at different magnifications, (b_2_, b_3_) delignified aspen wood at varying magnifications, (c_2,_ c_3_) TW at different magnifications, (d_2_) low vacuum SEM micrograph of R-TW without Pt/Pd surface coating, and (d_3_) high-resolution SEM micrographs of R-TW (insert: aggregates of CdSe/ZnS QDs).

The cellular structure of delignified wood (Figure 2b_2_) remains largely similar to that of native wood (Figure 2a_2_), while significant changes occur at the cell wall level. In particular, voids are formed in the middle lamella compound (MLC) and lignin-rich cell corner regions after lignin removal, as shown in Figure 2a_3_,b_3_. The mass loss after treatment is 17 ± 1%, primarily attributed to lignin extraction.

After impregnation with pre-MMA and subsequent polymerization, light scattering is significantly reduced due to improved refractive index matching between the wood cell wall (≈1.53) and the PMMA matrix (≈1.49), as evidenced by the increased transparency in Figure 2c_1_. SEM observations (Figure 2c_2_,c_3_) confirm that both the cell lumina and delignification-induced voids are effectively filled by the polymer. However, small interfacial gaps are observed at some cell wall-polymer interfaces, which are likely caused by polymerization shrinkage and limited interfacial adhesion.^15,32^

The R-TW composite (Figure 2d_1_) retains a similar level of transparency as neat TW, due to the low QD loading (≈0.07 mg/g). SEM images (Figure 2d_2_,2d_3_) show that QDs are primarily located within the polymer phase, particularly in the lumen regions. High-resolution imaging (inset in Figure 2d_3_) reveals the presence of nanoscale QD aggregates, indicating that dispersion is not fully uniform. This aggregation is attributed to limited miscibility between the QDs and the PMMA matrix.^33^ The CdSe/ZnS QDs are stabilized by relatively nonpolar ligands (e.g., oleic acid, oleylamine), which are not ideally matched to the more polar PMMA environment, leading to partial phase separation. Similar dispersion challenges have been reported in other polymer-QD systems.^34–36^

Alternative processing routes, such as preimpregnation of solvent-dispersed QDs into delignified wood prior to monomer infiltration, have been suggested.^21,24^ However, this approach resulted in significant QD loss and reduced quantum yield, likely due to the use of excess monomer required to minimize optical defects (e.g., voids). In addition, increased exposure to moisture and oxygen during processing may compromise QD stability and lead to luminescence degradation.^25,37^ Therefore, codispersion of QDs in the monomer solution followed by impregnation^2,12,25^ was preferred, as it improves QD retention and provides better protection against degradation during processing.

### 3.2 Tensile Properties

Mechanical properties are important for the structural integrity and practical implementation of TW-based photonic materials. Tensile tests were performed in the axial fiber direction. In Figure 3a, the stress–strain curves of neat PMMA and TW with a wood volume fraction (*V*_f_) of 20% are presented. TW shows ultimate strength of 154 MPa and Young’s modulus of 10.9 GPa. Comparable data for PMMA in Figure 3a are 44 MPa and 2.5 GPa, respectively. Based on a rule-of-mixtures analysis for TW, the effective modulus of the wood reinforcement in the fiber direction is 44.5 GPa. The mechanical enhancement is directly related to the *V*_f_, where increasing *V*_f_ leads to higher modulus (and strength) due to the high intrinsic modulus (and strength) of the wood cell wall structure.

**Figure 3:**
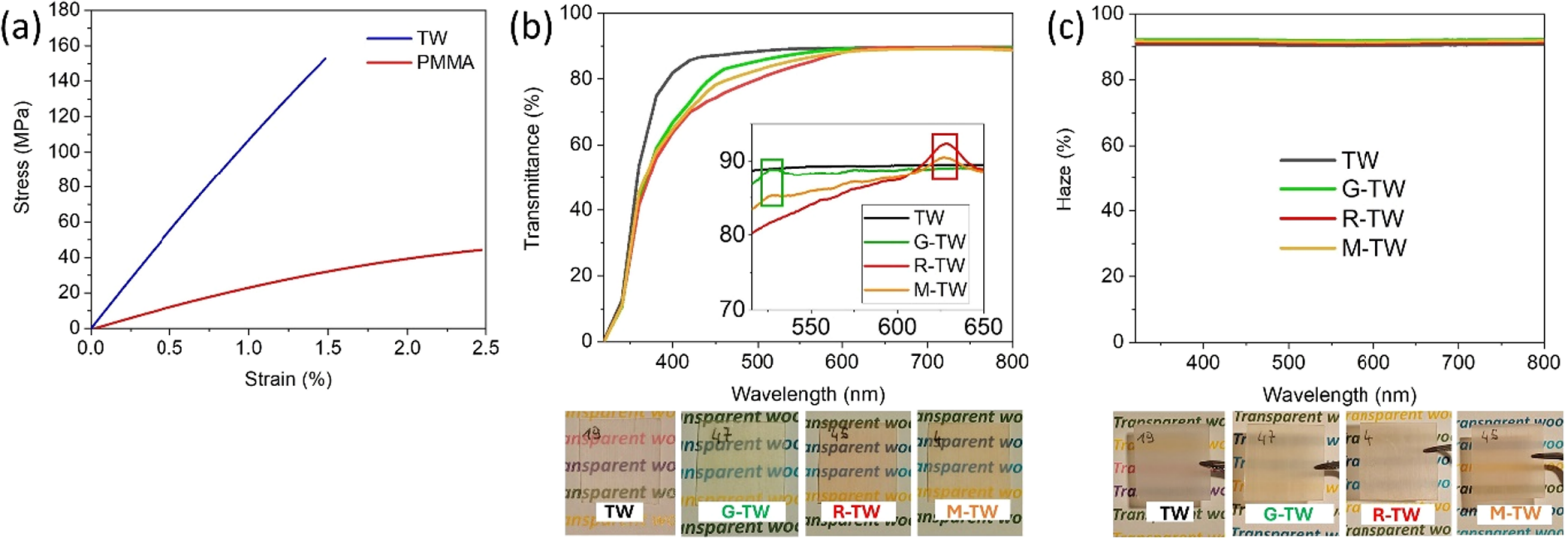
(a) Typical stress–strain curves in tension for neat PMMA and TW composites with a wood volume fraction of 20%. (b) Optical transmittance of 0.6 mm thick TW samples using discrete wavelengths (with an interval of 20 nm) of input light (inserted is the transmittance obtained using the input visible light wavelength at same time. Note: the data only shows the data between wavelength of 515 to 650 nm for a comparison). (c) haze of TW, G-TW, R-TW, M-TW. The respective macro images in (b, c) show the transmission and scattering phenomena.

### 3.3 Transmittance and Haze

Figure 3b shows the total transmittance (measured at discrete wavelengths from 320 to 800 nm) of TW, G-TW (green), R-TW (red), and M-TW (mixed red-green) with a wood substrate volume fraction *V*_f_ = 20% and a thickness of 0.6 mm. Transmittance is strongly influenced by wood volume fraction: increasing *V*_f_ leads to reduced transmittance due to enhanced light scattering within the composite.^19^ The increased scattering is either associated with scattering in the cell wall (Rayleigh and Mie scattering from “defects” smaller than the light wavelength), or with an increase in processing-induced optical defects. For transmittance at higher wavelengths (e.g., >600 nm), the transmittance for all materials is ≈89%. At shorter wavelengths, transmittance decreases for all QD-TWs since the QDs absorb light selectively at shorter wavelengths. The reason is larger density of states for transitions involving higher energy photons.^18,24,37^ Measurements of transmittance were also carried out under a broadband white light excitation, see insert in Figure 3b. For the QD-TW, there are small peaks in the green (525 nm) or red (625 nm) area, also presented in previous data.^24^ The peaks correspond to QD luminescence, which may be erroneously collected as transmitted light.

Figure 3c shows the haze values for all TW samples. All samples exhibit similarly high haze values (above 90%), suggesting that the incorporation of QDs does not significantly alter the scattering behavior, which is already dominated by the wood-polymer composite structure.

The high haze originates from air gaps/microcracks at wood-polymer interfaces as shown in Figure 2c, and refractive index mismatch between cell wall and PMMA, as well as additional scattering from structural features such as cell wall heterogeneity and nanoscale porosity.^38,39^ Thus, diffusely transmitted light^40^ was formed. The wood volume fraction plays a key role in this behavior: increasing *V*_f_ increases the density of scattering interfaces, leading to stronger light diffusion. In the present system (*V*_f_ = 20%), the haze is already close to saturation, indicating that further increases in *V*_f_ would have limited additional impact on haze but would continue to reduce transmittance. At the bottom of Figure 3 are photographs of TW, G-TW (green), R-TW (red), and M-TW (mixed) materials. Transparency is apparent when materials are directly on top of the text, but images become hazy due to scattered transmitted light as the distance from the sheet with the text is increased. This behavior reflects a fundamental trade-off in TW systems, where higher wood content improves mechanical performance but simultaneously increases optical scattering, thereby reducing light transmission.

### 3.4 Photoluminescence and Quantum Yield

Figure 4 presents the normalized photoluminescence (PL) spectra of TW composites containing CdSe/ZnS QDs under 450 nm excitation. The absorption contributions are separated into two components: absorption from the TW matrix without QDs (*A*_TW_, light blue area) and absorption from the QDs (*A*_QDs_, dark blue area). Based on this separation, the parasitic absorption contribution (η_parasitic_), defined as the fraction of light absorbed by nonactive components (wood substrate and PMMA matrix) that does not contribute to QD emission, can be quantified as

6 $$\eta_{\mathrm{parasitic}}=\frac{\int A_{\mathrm{TW}}\left(\lambda \right)d\lambda }{\int A_{\mathrm{TW}}\left(\lambda \right)d\lambda +\int A_{\mathrm{QDs}}\left(\lambda \right)d\lambda }$$

**Figure 4:**
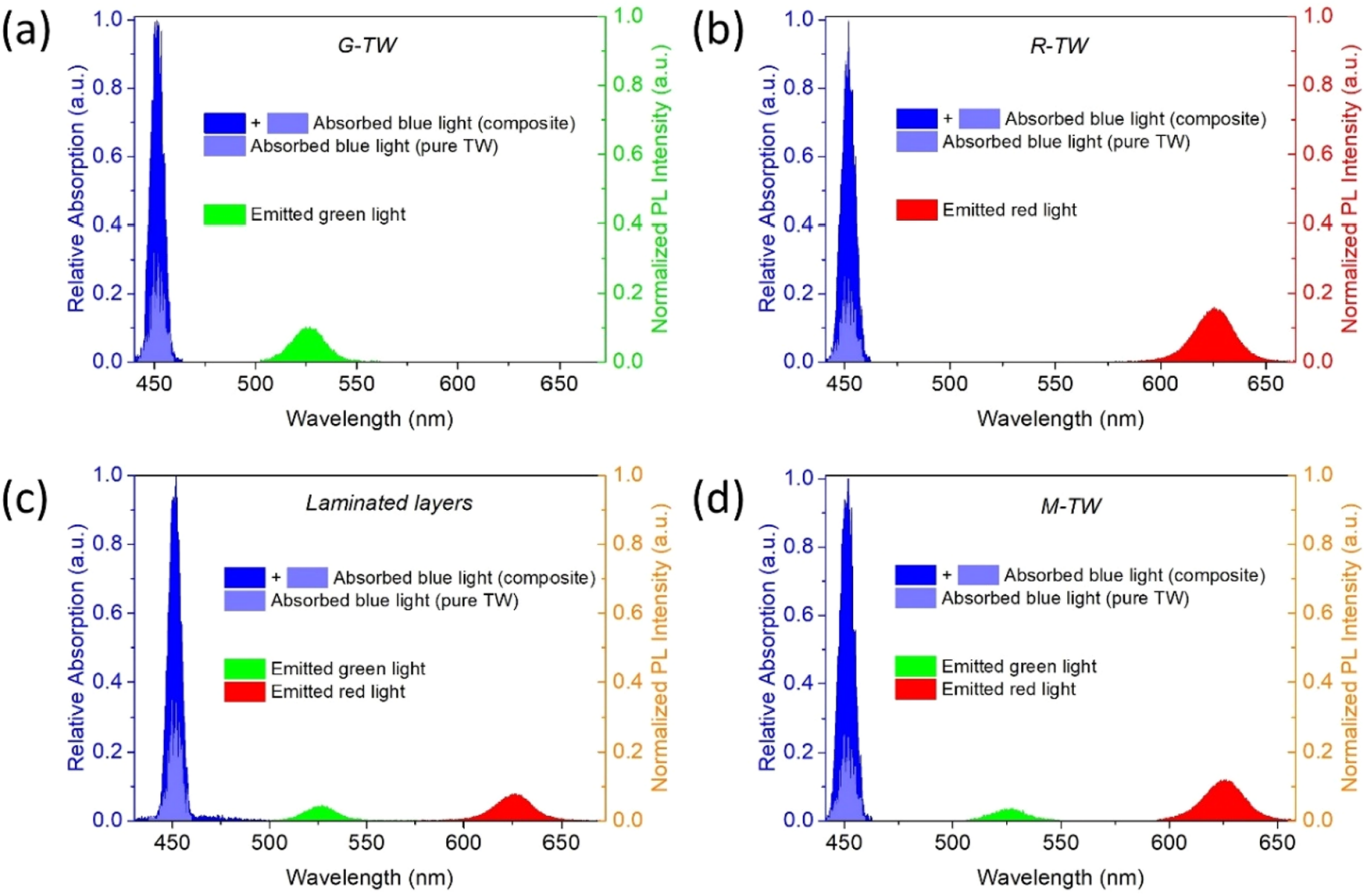
Normalized photoluminescence spectra (excited by 450 nm) of (a) green G-TW composites, (b) red R-TW composites, (c) two laminated layers (R-TW + G-TW), and (d) mixed green and red QDs in one M-TW composite.

Using this approach, η_parasitic_ values of 24.2% (G-TW) and 20.5% (R-TW) are obtained, indicating that a significant fraction of the excitation light is absorbed by the matrix rather than contributing to QD excitation. Correspondingly, the effective absorption by QDs is 75.8% and 79.5%, respectively. Similar values are observed for the laminated (L-TW, 25.2%) and mixed (M-TW, 19.5%) systems, confirming that the codispersion of QDs in a single layer does not introduce additional parasitic losses compared to layered structures.

The PL spectra in Figure 4a,4b show distinct emission peaks at 525 and 625 nm, corresponding to green and red CdSe/ZnS QDs, respectively. The combined spectra for laminated (Figure 4c) and mixed systems (Figure 4d) exhibit both emission peaks, demonstrating that effective spectral mixing is achieved within a single TW layer, comparable to multilayer configurations.

To further evaluate the PL efficiency, the quantum yield (QY) was calculated using two different approaches: (i) considering only the light absorbed by the QDs (excluding *A*_TW_), and (ii) considering the total absorbed excitation light by the entire composite (including *A*_TW_). At 450 nm excitation, the QD-based QY values are 36 ± 4% (G-TW), 67 ± 7.4% (R-TW), 53 ± 5.5% (L-TW), and 56 ± 5.8% (M-TW), as shown in Figure 5a–d. The M-TW composite shows comparable performance to the laminated structure, indicating that codispersion of QDs in a single layer does not compromise PL efficiency. When the total absorption of the composite is considered, the overall QY decreases to 27 ± 2.4% (G-TW), 53 ± 5.6% (R-TW), 37 ± 4% (L-TW), and 42 ± 4.5% (M-TW), due to parasitic absorption losses in the wood substrate and PMMA matrix that do not contribute to the emission. This reduction highlights the importance of minimizing matrix absorption to improve device efficiency.

**Figure 5:**
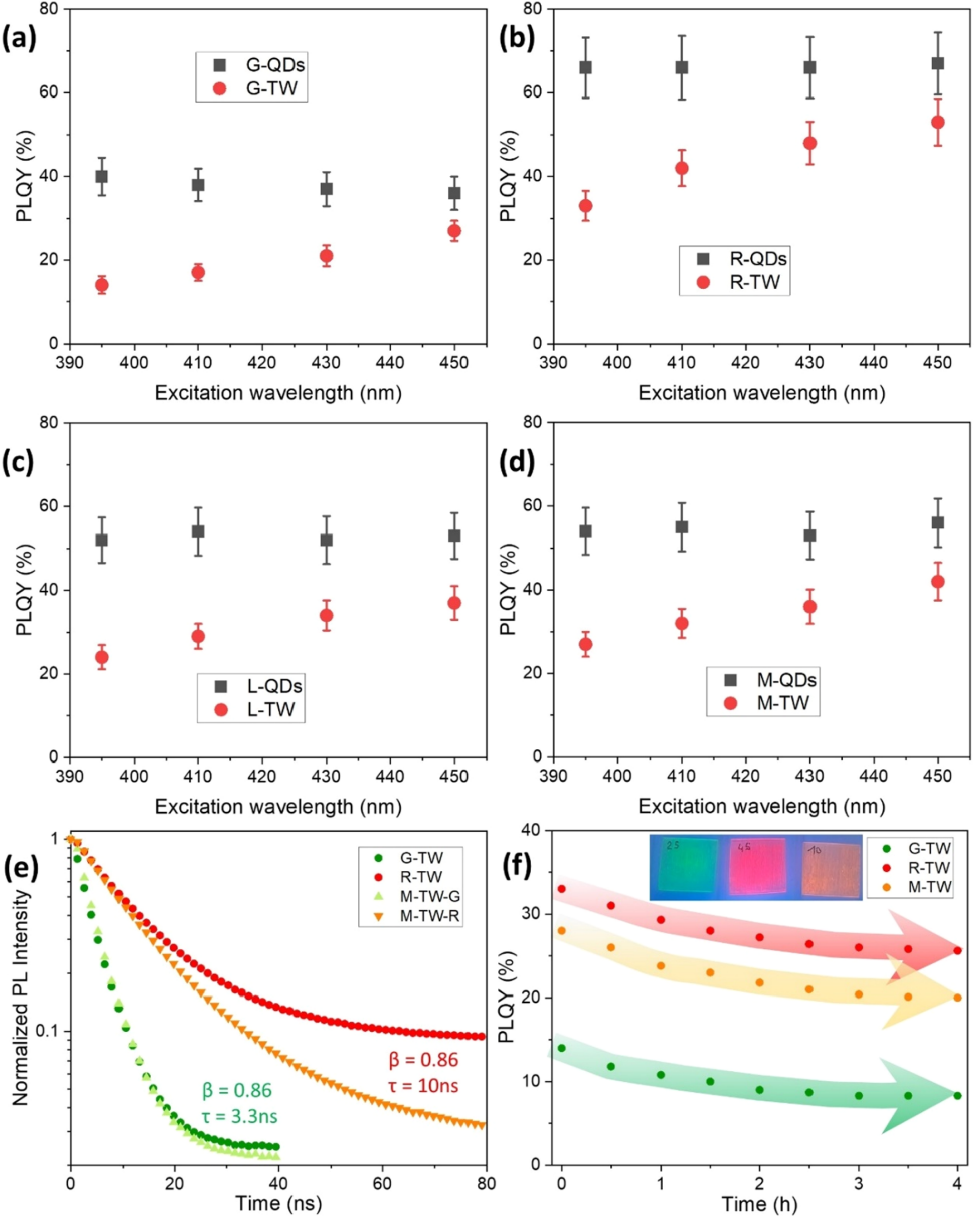
Quantum yield of (a) G-TW, (b) R-TW, (c) laminated 2 layers (L-TW), and (d) M-TW, at different excitation wavelengths, excluding (black dots) and considering (red dots) the absorbed blue light from the TW (without QDs) itself. (e) Time-resolved photoluminescence (TRPL) decay curves of G-TW, R-TW, and M-TW, where the green and red emissions of M-TW were measured separately. (f) Quantum yield of G-TW, R-TW, and M-TW under different UV exposure times (insert: photographs of the samples under UV illumination).

In addition, the QY in case (ii) shows a clear wavelength dependence, decreasing at shorter excitation wavelengths. This trend correlates with the increased parasitic absorption at lower wavelengths, as both PMMA and residual lignin exhibit stronger absorption in the UV-blue region (Figure 3b). Thus, η_parasitic_ is inherently wavelength-dependent and plays a critical role in determining the overall PL efficiency of the composite.

The QY values in the TW composites are significantly lower than those reported for QDs dispersed in toluene (>90% as reported from technical data sheet of the producer^37^), which is attributed to dispersion limitations in the solid matrix. Partial aggregation of QDs, as observed in SEM (Figure 2d), can introduce surface trap states and enhance nonradiative recombination pathways.^41^

To further understand the influence of QD dispersion and possible nonradiative recombination pathways in the QD-TW composites, time-resolved photoluminescence (TRPL) measurements were performed (Figure 5e). All samples exhibit nonsingle-exponential decay behavior, characteristic of QD-based solid-state systems. The decay curves were fitted using a stretched exponential model,^33,42^ yielding stretching factors (β) of approximately 0.86 for both green and red emissions, indicating relatively limited energetic disorder and a narrow distribution of recombination environments. The extracted characteristic lifetimes (τ) were approximately 3.3 ns for the green-emitting QDs and 10 ns for the red-emitting QDs in M-TW, consistent with the corresponding single-QD TW samples (G-TW and R-TW). This result suggests that codispersion of green and red QDs within a single TW layer does not introduce significant additional nonradiative recombination pathways. The lifetime values are markedly lower than for such QDs in the solution (10–20 ns for the green ones, and 20–30 ns for the red ones^43,44^ ). This result correlates well with the observed drop in QY for solid composites as new nonradiative transitions are introduced in the solid matrix. Nevertheless, the relatively low QY values of the TW composites compared with the solution-dispersed QDs suggest that multiple loss mechanisms are still present within the solid composite system. In addition to the nanoscale aggregation previously observed in SEM analysis (Figure 2d), parasitic absorption from the matrix (Figure 5a–d) and interface-related nonradiative relaxation processes may also contribute to photoluminescence losses. Furthermore, the highly scattering nature of the TW structure may increase local photon reabsorption and optical propagation losses within the composite. Further optimization of the QD dispersion within the solid matrix, for example through improved matrix compatibility, surface passivation, ligand modification, or optimization of the dispersion and curing processes, may provide an effective route to suppress aggregation-related quenching effects. Similar approaches, including ligand engineering and the use of surfactants, have previously been proposed to improve QD dispersion and mitigate aggregation-induced quenching.^25,33,45^

In addition to PL efficiency, long-term operational stability is also critical for practical optical applications. Therefore, the stability of the QD-TW composites under continuous UV excitation was investigated by monitoring the QY evolution over time (Figure 5f). All samples exhibit an initial decrease in QY during the first few hours of UV irradiation, followed by a relatively stable plateau at longer exposure times. Despite the initial reduction, the composites retain a considerable fraction of their emission efficiency after prolonged UV exposure, demonstrating reasonable photostability for optical applications under continuous excitation conditions.

Overall, these results demonstrate that parasitic absorption, QD dispersion, and nonradiative recombination processes are key factors governing the PL performance of TW-based composites, and should be carefully optimized in future materials design.

### 3.5 White Light Generation

Based on the optical properties discussed in the previous sections, including relatively high quantum yield, high optical transmittance, and strong light scattering (haze >90%), the QD-TW composites are well suited for color-conversion and diffuse light emission applications. The white light generation capability of the system is therefore evaluated.

Under UV-blue LED excitation (395 nm), the G-TW composite exhibits green emission (Figure 6a), originating from QD photoluminescence at 525 nm (Figure 4a). Due to the partial transmittance of the TW matrix, a fraction of the excitation blue light also passes through the sample, resulting in a mixed output composed of green emission and residual blue light. The corresponding CIE 1931 chromaticity^46^ coordinates (0.18, 0.51) confirm the green-dominated output (Figure 6g). A similar behavior is observed for R-TW (Figure 6b), where red emission at 625 nm (Figure 4b) is combined with transmitted blue light, yielding coordinates of (0.57, 0.27).

**Figure 6:**
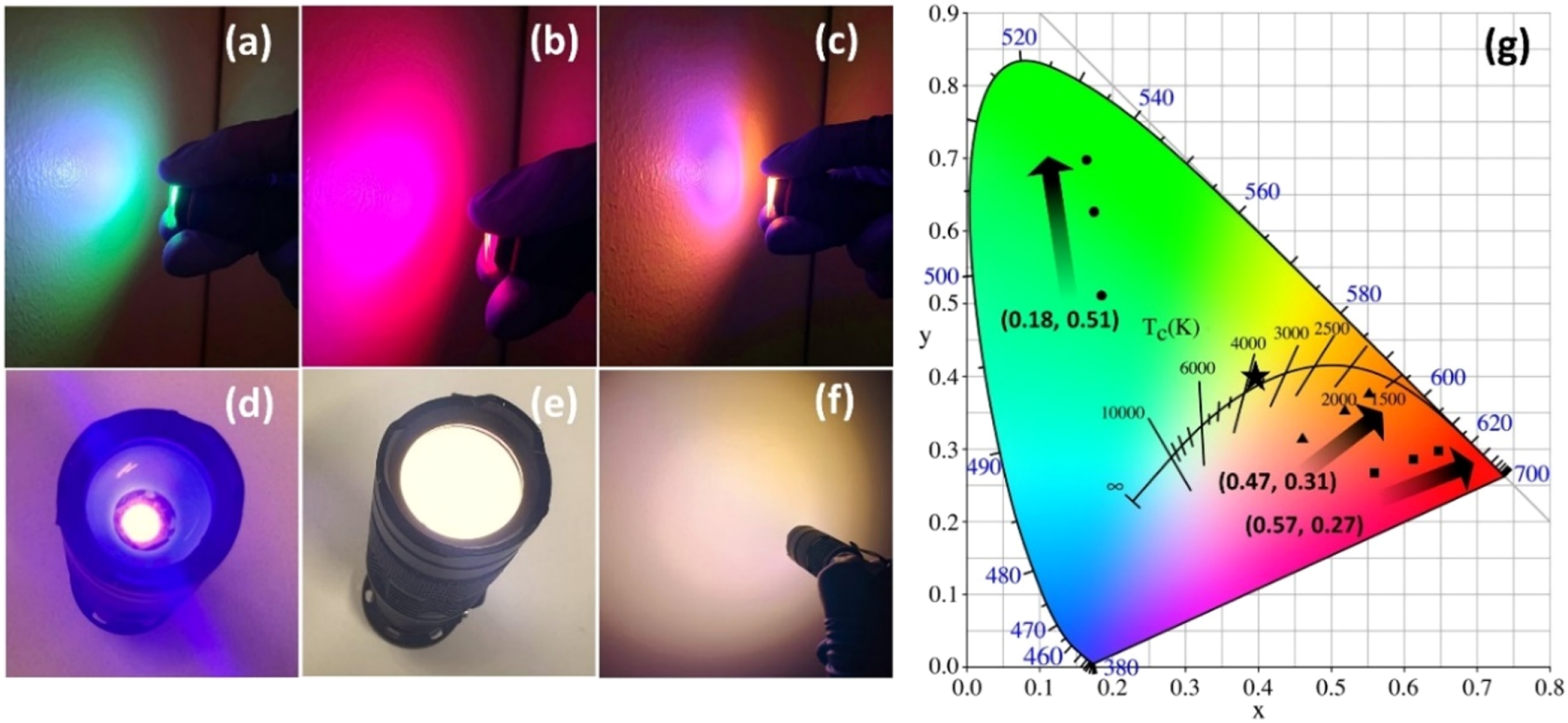
Images of transmitted light on a screen through (a) G-TW, (b) R-TW, and (c) M-TW using a 395 nm LED source. Image of this (d) LED source: a 395 nm UV torch, and (e) the M-TW composite used as a white light diffuser. (f) shows the projected transmitted white light on a screen, using the M-TW diffuser. (g) CIE 1931 color space showing the individual points corresponding to the transmitted colors of G-TW (circular dots), R-TW (squares), M-TW (triangles), and the white light diffuser (star).

For the M-TW composite containing both green and red QDs, the transmitted light exhibits an intermediate orange color (Figure 6c), as confirmed by the CIE coordinates (0.47, 0.31). This demonstrates effective spectral mixing of the two QD emissions within the TW matrix. In all three cases, decreasing the intensity of the excitation light reduces the transmitted blue component, resulting in more saturated green, red, or orange emission.

To achieve white light generation, the relative contributions of green and red QDs were adjusted. The relative amounts of the two QDs were estimated by considering both their relative emission intensity (*E*) in the M-TW composite (Figure 4d) and the wavelength-dependent sensitivity of the human eye.^47^ As a first approximation, the perceived brightness balance between green and red emission can be expressed as

7 $$W_{G}E_{G}S_{G}\approx W_{R}E_{R}S_{R}$$

where *W*_G_ and *W*_R_ are the weights of green and red QDs, *E*_G_ and *E*_R_ are their emission intensity in M-TW (*E*_G_/*E*_R_ ≈ 1:4.35), and *S*_G_ and *S*_R_ represent the photopic response of the human eye at the corresponding emission wavelengths (*S*_G_/*S*_R_ ≈ 7.5:3, at 525 and 625 nm,^47^ respectively). Based on this simplified relationship, a weight ratio of green to red QDs *W*_G_/*W*_R_ = 5:3 was obtained and used as a guideline for experimental design. It should be noted that the final white light output results from the combined contributions of red and green QD emission together with the transmitted blue excitation light, in addition to effects from absorption, scattering, and possible reabsorption within the composite.

The optimized M-TW sample was subsequently evaluated under the same excitation conditions (Figure 6d). The LED chip becomes visually obscured when covered by the composite (Figure 6e), indicating efficient light scattering and redistribution within the material. This leads to a more homogeneous emission with reduced glare and suppression of localized bright spots.

Transmitted white light is obtained (Figure 6f), and the corresponding CIE coordinates (0.39, 0.40) (Figure 6g) fall within the white light region, with a correlated color temperature (CCT) of approximately 3900 K. As discussed earlier, the relative balance between the transmitted blue excitation light and the QD emission can be adjusted by tuning the excitation intensity, QD loading, or the composite thickness, enabling modulation of both the CIE coordinates and the CCT of the emitted white light. The resulting white-light output is governed by the combined effects of excitation-light transmission, QD absorption and re-emission, multiple light scattering within the TW structure, and possible local reabsorption processes. Notably, unlike most previously reported luminescent TW systems that rely on either single-color QDs per layer or multilayer configurations for color mixing, the present approach employs codispersed green and red QDs within a single TW layer, where the transmitted excitation light simultaneously provides the blue component required for white light generation. This simplifies the material architecture while maintaining effective white light generation within the scattering medium. However, the strong scattering and high haze of TW may also introduce optical propagation losses and reduced direct transparency due to increased photon path lengths and local reabsorption effects within the composite. Therefore, an appropriate balance between haze and transmittance is important for optimizing both diffuse emission characteristics and overall optical efficiency.

Due to the narrow-band emission characteristics of the QDs, the generated white light spectrum remains partially discontinuous within certain regions of the visible range (Figure 4d), which may limit the achievable color rendering performance compared with broadband phosphor-based white light systems. Future improvements in color rendering may be achieved through broader spectral coverage and more continuous emission profiles by incorporating additional emissive components or broadband luminophores. Nevertheless, the present results demonstrate the feasibility of generating tunable white light from a single-layer QD-TW composite through spectral mixing between transmitted excitation light and QD emission. These findings highlight the potential of the proposed structure for lightweight luminescent optical applications.

## 4 Conclusions

Transparent wood (TW) composites with mixed red and green CdSe/ZnS quantum dots (QDs) in a single layer provide an efficient and structurally simple material for white light generation. The QDs were fairly uniformly distributed within the polymer-infiltrated wood matrix by premixing them in a pre-MMA solution prior to impregnation into the wood substrate. The TW composite shows an axial tensile strength of 154 MPa and a Young’s modulus of 10.9 GPa at 20 vol % wood content, and is suitable as a semistructural transparent light diffuser with integrated photonic functionality.

The QD-TW composites of 0.6 mm thickness show a relatively high photoluminescence quantum yield (42% for the mixed red-green system, M-TW), together with high optical transmittance (up to 89%) and significant haze (>90%). These combined optical properties enable efficient light diffusion and color conversion. By tuning the mixing ratio of green and red QDs, uniform and diffused white light emission was achieved under UV-blue LED excitation, with CIE coordinates (0.39, 0.40), located within the white-light region. The observed emission reflects a balanced spectral contribution from the mixed QDs, resulting in visually homogeneous white light with reduced glare.

Compared with conventional layered or single-emitter TW systems, the present approach demonstrates that codispersion of multiple QDs within a single TW layer enables straightforward fabrication with effective volumetric color mixing and integrated light management within a unified scattering medium. The present strategy eliminates the need for blue-emitting QDs, as the blue component of the white light is provided by the excitation source. The use of TW composites also provides opportunities for integrating optical functionality with aesthetic and structural design.
